# Letter to Editor “Comments on self-medication with conventional and herbal medicines in pregnancy: prevalence and factors in Northwest Ethiopia”

**DOI:** 10.1097/MS9.0000000000004980

**Published:** 2026-05-01

**Authors:** Bisma Bashir Ahmed, Imad Khan, Sangeen Khan, Adeenah Irshad, Abdul Haseeb Hasan

**Affiliations:** a Department of Medicine, People’s University of Medical and Health Sciences for Women, Nawabshah, Pakistan; b Department of Medicine, Northwest School of Medicine, Peshawar, Pakistan; c Department of Medicine, Bolan Medical College, Quetta, Pakistan; d Department of Medicine, Liaquat University of Medical and Health Sciences, Jamshoro, Pakistan; e Department of Research, Oli Health Magazine Organization, Research and Education, Kigali, Rwanda; f Department of Medicine, King Edward Medical University, Lahore, Pakistan; g Department of Internal Medicine, Sentara Northern Virginia Medical Center, VA, USA

*Dear Editor,*

We read with great interest the article by Feyisa *et al* titled[1] “Self-medication with conventional and herbal medicines in pregnancy: prevalence and factors in Northwest Ethiopia,” published in Annals of Medicine & Surgery. The authors deserve to be appreciated for addressing the understudied issue of self-medication during pregnancy in a low-resource setting, yet some gaps in the methodology and interpretation need to be addressed.

In this study, data were gathered through face-to-face interviews conducted by pharmacy specialists immediately following antenatal care visits, a context likely conducive to the underreporting of stigmatized behaviors. The disproportionate number of women, who give the causes of abstinence as reasons of fear of causing an abortion (84.5%), or provider advice (77.5%), is an indication that they may have over-reported non-use. There were no anti-bias methods used, such as anonymity or validated instruments. This likely led to an underestimation of both conventional and herbal medicine practices. Past researches have indicated that this bias may be minimized by anonymous questionnaires and by avoiding immediate post-consultation settings[2].

A notable methodological gap is the absence of pregnancy safety classification of the reported medicines. Although the study identifies which drugs and herbs were used, it does not categorize them according to established pregnancy safety frameworks such as the U.S. Food and Drug Administration (FDA) pregnancy risk categories or equivalent systems. By contrast, a cross-sectional study in Ethiopia explicitly applied FDA pregnancy risk categories to the reported drugs and found that 13.6% of self-medicated women had used potentially risky agents (category C, D, or X). Notably, the authors of the current study define the FDA drug classification system in their own operational definitions section, yet never apply it to their findings, a significant missed opportunity that considerably limits the public health implications of this work[3].

The study also fails to capture data on the dose, duration, and frequency of self-medication, which are critical determinants of fetal risk. Knowing that 77.0% of women used paracetamol is important, but the clinical risk of paracetamol in pregnancy is dose- and duration-dependent, a single dose for acute headache carries a different risk profile than prolonged daily use, which has been associated with adverse neurodevelopmental outcomes in the offspring[4]. Similarly, the herbal medicines reported, particularly Ruta chalepensis (Tenaadam, used by 31.7%) have documented uterotonic and abortifacient properties at higher doses, yet the study does not quantify how much was consumed or for how long. In comparable studies, such as a cross-sectional study in Ethiopia, dose and duration data were collected alongside the medicines used, enabling a more complete assessment of potential harm[5]. The omission of dosing information in the current study means that the reported prevalence figures cannot be translated into an estimate of clinical risk at the individual or population level[6].

## Data Availability

Data sharing not applicable to this article as no datasets were generated or analyzed during the current study.

## Declaration of generative Al and Al-assisted technologies in the writing process

There is nothing to disclose.

## References

[1] Feyisa K Kebede SY Mekonnen BA. Self-medication with conventional and herbal medicines in pregnancy: prevalence and factors in Northwest Ethiopia. Ann Med Surg 2026;88:1275–86 10.1097/MS9.0000000000004613 PMC12889330 41675894

[2] Jovanović Ž Vulić P. Self-medication and knowledge of pregnant women about the use of medication during pregnancy in the cities of Rijeka and Zadar, Croatia. Front Pharmacol 2025;16:1536050 39925844 10.3389/fphar.2025.1536050 PMC11803312

[3] Ahmed SM Sundby J Aragaw YA. Self-medication and safety profile of medicines used among pregnant women in a tertiary teaching hospital in Jimma, Ethiopia: a cross-sectional study. Int J Environ Res Public Health 2020;17:3993 32512804 10.3390/ijerph17113993 PMC7312933

[4] Alemany S Avella-García C Liew Z. Prenatal and postnatal exposure to acetaminophen in relation to autism spectrum and attention-deficit and hyperactivity symptoms in childhood: meta-analysis in six European population-based cohorts. Eur J Epidemiol 2021;36: 993–1004 34046850 10.1007/s10654-021-00754-4 PMC8542535

[5] Niriayo YL Mohammed K Asgedom SW. Self-medication practice and contributing factors among pregnant women. Bourgeois D, ed. PLOS ONE 2021;16:e0251725 34014975 10.1371/journal.pone.0251725 PMC8136661

[6] Kennedy D Batagol R. Drug safety in pregnancy. Aust Prescr 2025;48:5–9 40040742 10.18773/austprescr.2025.008 PMC11875727

